# Cystitis Cystica et Glandularis: A Dual Case Report and Review of Contemporary Management

**DOI:** 10.7759/cureus.111715

**Published:** 2026-06-29

**Authors:** Sumiyya Rashid, Simranjot Kaur

**Affiliations:** 1 Urology, University Hospitals of Leicester NHS Trust, Leicester, GBR

**Keywords:** bilateral hydronephrosis, cystitis cystica, cystitis glandularis, lower urinary tract symptoms, malignancy risk, multidisciplinary team (mdt), recurrent urinary tract infections, renal function, renogram, transurethral resection of bladder tumour (turbt)

## Abstract

Cystitis cystica et glandularis (CCG) is a benign bladder condition driven by chronic mucosal irritation. Although often incidental, extensive disease can cause distal ureteric obstruction and produce radiological and cystoscopic appearances that closely mimic bladder malignancy, creating significant diagnostic and management challenges.

We report two men with histologically confirmed CCG whose clinical courses differed markedly. The first presented with bilateral hydronephrosis and bladder distortion, initially suspicious for urothelial carcinoma. Despite repeated resections, stenting and nephrostomy drainage, obstruction persisted and bilateral ureteric reimplantation was ultimately required to preserve renal function. The second presented with recurrent urinary infections and voiding dysfunction refractory to antibiotics. Despite bilateral hydronephrosis, mercaptoacetyltriglycine (MAG3) renography excluded true obstruction. His symptoms reflected a non-prostatic outflow problem, and conservative management was pursued; however, with ongoing symptoms, cystectomy remains under consideration.

These cases highlight that clinical outcomes are determined primarily by the level and functional significance of obstruction, rather than histological subtype alone, and that early multidisciplinary assessment and individualised management are key to minimising morbidity.

## Introduction

Cystitis cystica et glandularis (CCG) comprises a group of benign proliferative lesions of the bladder mucosa arising from chronic urothelial irritation. Recognised triggers include recurrent urinary tract infections (UTIs), urolithiasis, bladder outlet obstruction and other mechanical insults [1]. Histologically, cystitis cystica results from cystic dilatation of von Brunn's nests, whereas cystitis glandularis reflects glandular urothelial metaplasia [1]. Two subtypes exist: the typical (non-intestinal) form and the intestinal metaplasia (IM) variant, the latter characterised by prominent glandular proliferation, mucin production and a predilection for the bladder neck and trigone [1,2]. 

CCG poses a diagnostic challenge, as its cystoscopic and radiological appearance can closely resemble bladder carcinoma. Its true prevalence is likely underestimated, given that most cases are asymptomatic and identified incidentally [1]. Although generally considered benign, the malignant potential of the IM subtype remains contested due to histological overlap with adenocarcinoma and high-grade urothelial carcinoma [3,4]. Careful histopathological assessment is therefore essential to exclude neoplasia and guide management. We report two male patients with extensive CCG who followed markedly different clinical courses, illustrating the importance of integrating anatomical findings with functional outcomes.

## Case presentation

A 46-year-old male was referred in January 2020 following incidental imaging revealing bilateral hydronephrosis. He was asymptomatic with normal renal function. Flexible cystoscopy demonstrated an abnormal bladder neck and trigone with poor visualisation of both ureteric orifices. CT urogram raised concern for bladder malignancy (T3bN1M0) (Figures 1, 2). Progressive obstruction necessitated right ureteric stenting and left nephrostomy after failed retrograde stenting. Transurethral resection of bladder tumour (TURBT) confirmed cystitis glandularis with IM, and a mercaptoacetyltriglycine (MAG3) renogram demonstrated preserved, equally split renal function.

**Figure 1:**
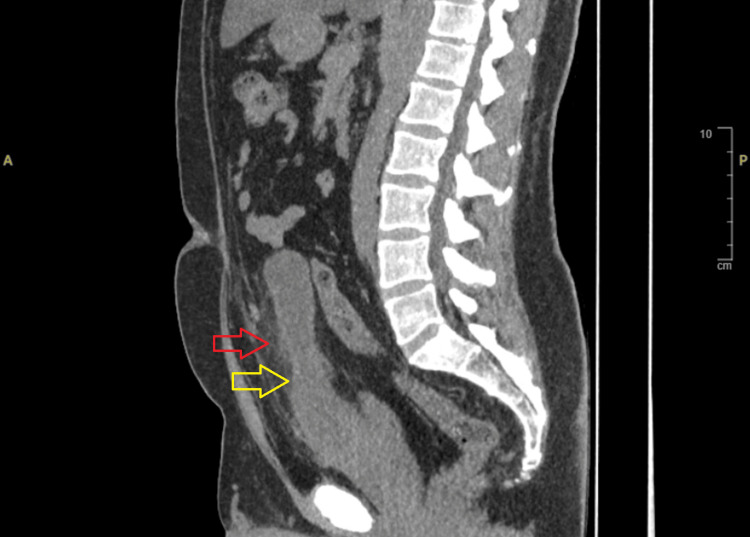
CT urogram (2020) - Initial imaging raising concern for urothelial malignancy Sagittal CT urogram demonstrating an underdistended bladder with anterior wall thickening (yellow arrow) and surrounding perivesical stranding (red arrow).

**Figure 2:**
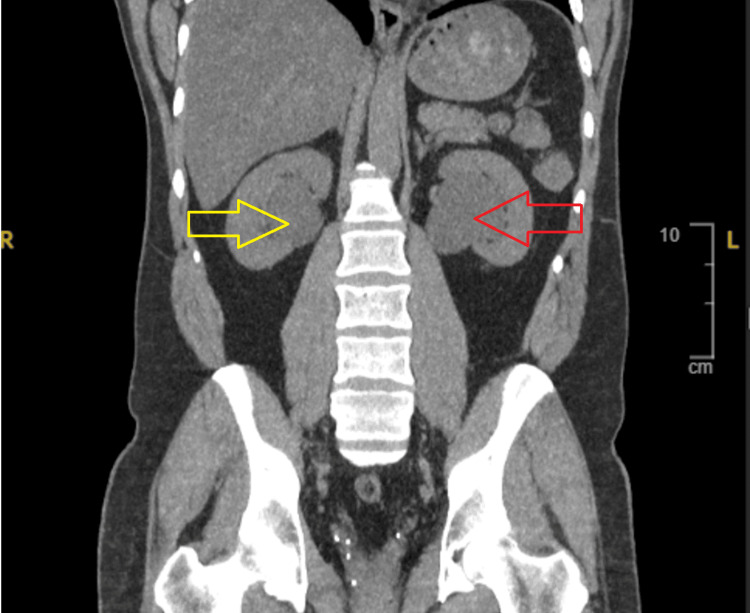
CT urogram (2020) - Bilateral hydronephrosis Coronal CT urogram demonstrating marked dilatation of the right renal collecting system (yellow arrow) and left renal collecting system (red arrow), reflecting bilateral obstructive uropathy secondary to bladder wall disease.

Despite benign histology, his course was complicated by haematuria, clot retention and urosepsis, necessitating bilateral nephrostomies. Repeat TURBTs confirmed cystitis glandularis. Urodynamics excluded bladder outlet obstruction, and MRI demonstrated distal ureteric obstruction without progression, extrinsic compression or pelvic lipomatosis. Methenamine hippurate was commenced. Given an isolated upper tract obstruction without an outflow component, the multidisciplinary team (MDT) consensus favoured definitive intervention. He underwent bilateral ureteric reimplantation in July 2021 with nephrostomy removal. Post-operatively, he developed acute kidney injury requiring temporary left nephrostomy reinsertion. Subsequent ultrasound and MAG3 renography surveillance demonstrated stable upper tract dilatation with preserved drainage. By March 2025, renal function remained stable (estimated glomerular filtration rate (eGFR) 81 mL/min/1.73 m^2^), and the patient remains under annual clinical review.

The second case was a 53-year-old male who initially presented with recurrent UTIs and lower urinary tract symptoms, including poor urinary flow. Antibiotics and solifenacin were ineffective during his clinical course. Ultrasound demonstrated bilateral hydronephrosis and a 22 mm posterior bladder cystic lesion, initially raising suspicion for a ureterocele. Flexible cystoscopy showed a distended bladder with difficulty visualising the left ureteric orifice. CT urogram demonstrated bladder wall thickening at the ureteric insertion sites with bilateral ureteric tapering, suggesting functional obstruction (Figure 3). Video urodynamics supported bladder outlet obstruction of unclear aetiology, not attributable to prostatic enlargement. MAG3 renography showed slow bilateral drainage without mechanical obstruction, and pelvic MRI excluded extrinsic compression and pelvic lipomatosis, confirming no true upper tract obstruction.

**Figure 3:**
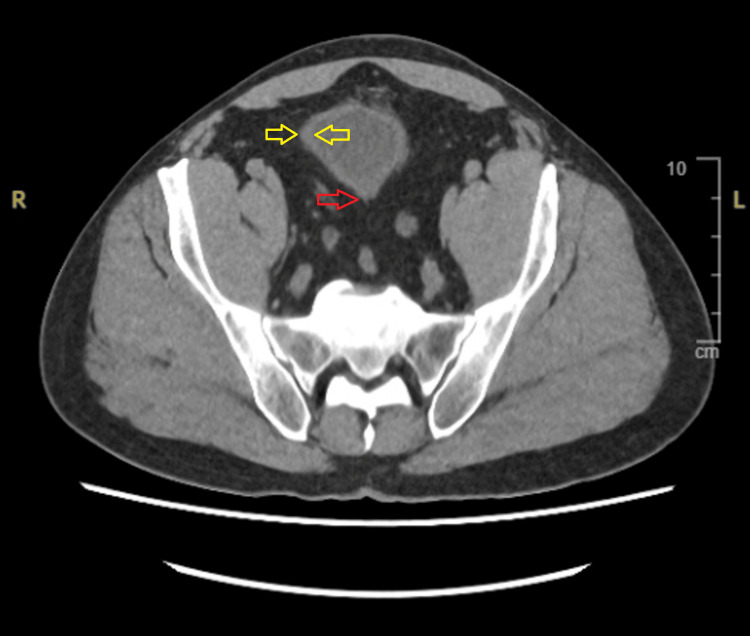
CT urogram (2024) - Imaging confirming inflammatory bladder changes Axial CT urogram demonstrating thickened, trabeculated bladder wall at the base (yellow arrows) with irregular bladder contour (red arrow).

Histology demonstrated extensive cystitis glandularis with complete IM. Renal function was preserved (eGFR 83 mL/min/1.73 m^2^) and, in the absence of true obstruction, MDT review recommended biannual surveillance. Repeat TURBT at six months showed no disease progression. Upper tract dilatation persists on surveillance imaging, though renal function remains stable. Given ongoing symptoms attributable to a non-prostatic outflow problem, cystectomy is under consideration.

## Discussion

CCG frequently creates diagnostic uncertainty, as cystoscopic and radiological appearances can closely resemble malignancy. Reported endoscopic findings demonstrate nodular, polypoid and cobblestone changes, and imaging may demonstrate bladder wall thickening, ureteric tapering and hydronephrosis [1]. These features seen on endoscopy and imaging are often indistinguishable from invasive carcinoma [3]. CT overstaging, as occurred in Case 1 (T3bN1M0), reflects a recognised limitation of cross-sectional imaging. Histopathology, therefore, remains essential; cystic dilatation of urothelial nests confirms CCG, and the absence of stromal invasion excludes malignancy [1]. Although the intestinal subtype has historically raised concern for malignant transformation, contemporary evidence suggests progression is rare even in extensive disease [3,4]. Immunohistochemistry tests, such as CK7, CK20 and CDX-2, are not routinely required but may assist in more challenging cases [5]. CCG may also mimic pelvic lipomatosis on imaging; CT and MRI can help differentiate between the two, with pelvic lipomatosis characteristically demonstrating extrinsic perivesical fat proliferation and a pear-shaped bladder deformity with bilateral ureteric obstruction [6].

Management is guided by symptoms and functional impact rather than oncological risk. Stable disease may be managed conservatively with surveillance and optimisation of urinary health and voiding function. Recurrent infections have been managed with antibiotic prophylaxis, D-mannose or intravesical sodium hyaluronate, though evidence remains limited [1,5]. Methenamine hippurate offers an antimicrobial-sparing alternative, as illustrated by infection-free stability in Case 1 during surgical reconstruction. Unlike urothelial carcinomas, there is no evidence to support intravesical BCG (Bacillus Calmette-Guérin) nor systemic immunotherapy in the management of CCG. In refractory cases, corticosteroids or COX-2 inhibitors have been employed, reinforcing the concept of CCG as a chronic inflammatory condition [1,5].

Persistent obstruction, infection or renal impairment may necessitate surgical intervention. TURBT remains both diagnostic and therapeutic [1,5]. Bladder-sparing reconstruction may be appropriate where function is preserved, as illustrated in Case 1. Although prophylactic ureteric reimplantation is not routinely recommended, delayed intervention risks prolonged decompression and cumulative morbidity from distorted anatomy. Conversely, Case 2 illustrates that in the absence of true upper tract obstruction with non-prostatic bladder outlet obstruction, conservative management may be justified despite ongoing symptoms, with cystectomy reserved for severe refractory disease [1,3]. 

No standardised surveillance protocol exists. Six- to 12-monthly cystoscopy with upper tract imaging has been proposed for higher-risk patients, particularly those with trigonal involvement or prior obstruction [3,5]. Surveillance should be individualised, guided by symptoms, renal function and functional assessment. Renal ultrasound, selective renography and periodic renal function testing help distinguish dilatation from true obstruction. MDT follow-up is essential to ensure timely escalation in patients with evolving functional compromise while avoiding unnecessary intervention in stable disease.

## Conclusions

In summary, CCG is a benign but clinically significant condition that frequently mimics malignancy, leading to diagnostic uncertainty and repeated intervention. Clinical outcomes are determined primarily by the level and functional significance of obstruction, rather than histological subtype alone. Management should therefore be function-directed, focusing on relieving underlying irritation, controlling infection and protecting renal function, with surveillance appropriate in stable disease. Reconstructive procedures, ranging from TURBT to cystectomy, may be required in extensive obstructive cases. 

Both cases highlight the importance of distinguishing true obstruction from compensated or non-obstructive hydronephrosis using serial renal function testing and functional imaging such as MAG3 renography. Management decisions were guided by considerations of bladder compliance and renal preservation, which supported a bladder-sparing approach. Early MDT involvement, integration of anatomical and functional assessment, and individualised surveillance may help minimise morbidity and support long-term renal outcomes.

## References

[1] Abdel Magied MH, Badreldin AM, Leslie SW (2024). Cystitis cystica and cystitis glandularis. StatPearls [Internet].

[2] Zhang T, Yin SF, Feng WB, Ke CX (2023). Florid cystitis glandularis (intestinal type) with mucus extravasation: Two case reports and literature review. Front Surg.

[3] Khan N, Abu Ghanem Y, Ng W, Nair R, Mensah E, Thurairaja R, Khan MS (2025). Cystitis glandularis: A 10-year institutional experience and review of the literature. Cureus.

[4] Agrawal A, Kumar D, Jha AA, Aggarwal P (2020). Incidence of adenocarcinoma bladder in patients with cystitis cystica et glandularis: A retrospective study. Indian J Urol.

[5] Oukouhou A, Harchaoui MA, Ameur Y (2026). Recurrent cystitis glandularis of the bladder: Case report and review. Urol Case Rep.

[6] Masumori N, Tsukamoto T (1999). Pelvic lipomatosis associated with proliferative cystitis: Case report and review of the Japanese literature. Int J Urol.

